# Impact of nonpharmaceutical interventions on infectious disease patterns in Yunnan Province across the COVID-19 pandemic phases

**DOI:** 10.1186/s12879-025-12386-0

**Published:** 2026-01-08

**Authors:** Lihua Chen, Huxing Gao, Jiarui Zhang, Chuanzhi Xu, Shuiping Lu, Yue Pan, Karuppiah Thilakavathy, Shiwen Zhao, Chenglong Xiong, Zhong Sun

**Affiliations:** 1 School of Public Health, Kunming Medical University, Kunming, 650500 China; 2 Yunnan Provincial Center for Disease Control and Prevention, 158 Dongsi Street, Kunming, 650022 China; 3 School of Public Health, Haiyuan College, Kunming Medical University, Kunming, 650106 China; 4 Key Lab of Public Health Safety, School of Public Health, Fudan University, Ministry of Education, Shanghai, 200433 China; 5 Department of Biomedical Sciences, Faculty of Medicine and Health Sciences, Universiti Putra Malaysia, Serdang, 43400 Malaysia

**Keywords:** COVID-19 pandemic, Infectious disease trends, Nonpharmaceutical interventions (NPIs), Yunnan province, Disease rebound, Vector-borne infections, Zoonoses, WaSH, Postpandemic surveillance

## Abstract

**Background:**

The COVID-19 pandemic substantially altered global infectious disease patterns. Nonpharmaceutical interventions (NPIs)—including lockdowns, travel restrictions, and enhanced hygiene—were widely implemented to control SARS-CoV-2 but also influenced the transmission of other infectious diseases. This study assessed the impact of NPIs on 36 notifiable diseases in Yunnan Province, China, across three phases: the prepandemic period (2014–2019), the pandemic phase (2020–2022), and the postpandemic phase (2023), when most NPIs were lifted.

**Methods:**

We analyzed surveillance data from the Yunnan Provincial CDC using Poisson regression, Spearman correlation, and bootstrap resampling. Incidence ratios and temporal trends were compared across phases, adjusting for population size. All analyses were performed using R.

**Results:**

During the pandemic phase, NPIs significantly suppressed several diseases, including leptospirosis (− 97.4%, *P* < 0.001), rabies (− 90.2%, *P* < 0.001), and schistosomiasis (− 88.9%, *P* < 0.05), likely due to reduced mobility and improved hygiene. In contrast, brucellosis (+ 195%, *P* < 0.001) and pertussis (+ 559.3%, *P* < 0.001) increased, potentially due to service disruptions. After NPI relaxation in 2023, marked rebounds were observed in dengue fever (+ 5027.4%, *P* < 0.001) and influenza (+ 1304.3%, *P* < 0.001), likely reflecting waning population immunity. However, sustained reductions persisted in tuberculosis (− 12.5%, *P* < 0.001) and rubella (− 89.3%, *P* < 0.001).

**Conclusions:**

NPIs, especially travel restrictions and improved hygiene, effectively curtailed the transmission of multiple infectious diseases in Yunnan during the pandemic phase. The postpandemic resurgence of several diseases underscores the importance of sustained surveillance and context-tailored public health strategies to maintain disease control and strengthen system resilience.

**Supplementary Information:**

The online version contains supplementary material available at 10.1186/s12879-025-12386-0.

## Introduction

The COVID-19 pandemic, a major global health crisis of the 21st century, caused substantial morbidity and mortality through the spread of SARS-CoV-2 and led to widespread changes in infectious disease epidemiology. Nonpharmaceutical interventions (NPIs)—including social distancing, mask mandates, enhanced hygiene practices, and travel restrictions—were rapidly implemented to reduce viral transmission in the absence of vaccines. While primarily aimed at controlling COVID-19, these interventions also disrupted the transmission patterns of other communicable diseases by altering human contact behavior, mobility, and healthcare utilization. As a result, the pandemic provided a natural quasi-experimental setting to examine how NPIs may influence the patterns and dynamics of a broad spectrum of infectious diseases [1, 2].

Yunnan Province in southwestern China provides an informative setting to study these effects. The region borders Southeast Asia and exhibits high ecological diversity, which supports endemic transmission of multiple infectious diseases—including vector-borne (e.g., dengue, malaria), zoonotic (e.g., brucellosis), and respiratory infections (e.g., tuberculosis). Furthermore, the province benefits from a robust and continuous disease surveillance system under the national Infectious Disease Control Law, offering reliable longitudinal data on notifiable conditions, enabling analyses across different pathogen types and transmission pathways.

This study analyzes temporal trends in monthly reported case counts for 36 infectious diseases in Yunnan across three distinct phases: the prepandemic period (2014–2019), when no COVID-19-related interventions were in place; the pandemic phase (2020–2022), marked by the implementation of stringent NPIs; and the postpandemic adjustment phase (2023), during which most NPIs were relaxed. By comparing monthly case patterns across these intervals, we aim to assess the associations between NPI implementation and shifts in disease burden, as well as the extent and trajectory of disease resurgence following policy relaxation. This observational study does not attempt causal inference but rather seeks to characterize syndemic transitions and provide region-specific insights for postpandemic public health planning [3, 4].

Globally, NPIs have been shown to reduce respiratory, waterborne, and contact-transmitted diseases, while their withdrawal has led to sharp resurgences in certain infections, particularly those with ecological or behavioral vectors [5, 6]. Similar trends have also been reported in China, where interventions such as mass testing and travel restrictions significantly reduced the burden of diseases like tuberculosis and enteric infections during the pandemic [5]. By contextualizing these trends within Yunnan Province, this study contributes to a growing body of evidence on the extended impacts of NPIs and offers insight into sustainable, region-specific disease control strategies in the post-COVID era.

## Methods

### Data sources and endemic levels of infectious diseases in Yunnan, China

We utilized surveillance data for 45 infectious diseases reported in Yunnan Province between January 2014 and December 2023, obtained from the Yunnan Provincial Center for Disease Control and Prevention (CDC). This dataset captured weekly case counts, compiled under China’s Infectious Disease Control Law, encompassing both notifiable diseases and sentinel-monitored conditions. Diagnostic approaches included polymerase chain reaction (PCR) testing, antigen assays, and clinical diagnosis, with consistent diagnostic protocols maintained across the full observation period. Laboratory confirmation was available for all notifiable diseases and selected sentinel conditions [7].

To prepare the data for analysis, weekly reports were aggregated into monthly case counts by summing values across calendar weeks. Where calendar weeks spanned two months, the week was assigned to the month with the majority of days. Missing weekly data points, if any, were treated as zeros to maintain consistency in aggregation. During preprocessing, six diseases—filariasis, diphtheria, poliomyelitis, severe acute respiratory syndrome (SARS), cholera, and plague—were excluded due to the complete absence of reported cases throughout the study period. Likewise, H7N9 avian influenza and highly pathogenic avian influenza were excluded due to extremely low case counts and the absence of reported cases after 2020, which precluded meaningful trend analysis. These exclusions were made solely for analytical feasibility and do not affect the overall assessment of population-level disease dynamics.

The final dataset included 36 infectious diseases with sufficient temporal continuity and case volume to support robust statistical analyses of pandemic-phase and postpandemic-phase shifts in reported disease burden.

To facilitate phase-based comparisons, the study period was categorized into three intervals: (1) Prepandemic phase (2014–2019): Baseline disease patterns before COVID-19 emergence. (2) COVID-19 pandemic phase (2020–2022): Characterized by implementation of stringent NPIs, including lockdowns, travel restrictions, mass testing, and school closures. (3) Postpandemic adjustment phase (2023): Marked by the nationwide downgrading of COVID-19 to a Category B infectious disease and the widespread relaxation of control measures, including the lifting of mandatory quarantine and travel bans.

Although the pandemic phase is treated as a single analytical unit, internal variation (e.g., between stricter and more relaxed NPI periods) was also examined descriptively in downstream analyses.

In 2023, the full reopening of society, resumption of large-scale mobility, and return to pre-pandemic behaviors provided a natural opportunity to observe rebound or stabilization patterns in infectious disease trends. Phase-specific average monthly case counts and percentage changes across all diseases are presented in Table 1, and temporal trends over the 10-year period are illustrated in Supplementary Fig. 1.

**Table 1:** Epidemiological characteristics and comparison of mean annual reported cases for 36 infectious diseases across the prepandemic (2014–2019), COVID-19 pandemic phase (2020–2022), and postpandemic phase (2023)

Infectious disease	Causative pathogen	Main mode of transmission	Mean number of reported cases			Percent difference		
			2014–2019	2020–2022	2023	2020–2022 vs. 2014–2019	2023 vs. 2014–2019	2023 vs. 2020–2022
Hand-foot-and-mouth disease	*Enterovirus 71*,* Coxsackievirus*	Direct contact transmission	88205	68897	66250	-21.9***	-24.9***	-3.8***
Other infectious diarrheal diseases	Various pathogens (e.g., *E. coli*,* Salmonella*)	Fecal-oral transmission	28180	40749	49150	44.6***	74.4***	20.6***
Echinococcosis	*Echinococcus granulosus*,* E. multilocularis*	Ingestion of contaminated food/water	12	23	31	91.7	158.3**	34.8
Visceral Leishmaniasis	*Leishmania spp.*	Vector-borne (sandfly bites)	1	3	1	200	-1.1	-66.7
Typhus	*Rickettsia spp.*	Vector-borne (lice or fleas)	431	405	492	-6	14.1*	21.5**
Leprosy	*Mycobacterium leprae*	Prolonged close contact	173	95	88	-45.1***	-49.1***	-7.4
Acute Hemorrhagic Conjunctivitis	*Enterovirus 70*,* Coxsackievirus A24*	Direct contact transmission	2648	1144	4250	-56.8***	60.5***	271.5***
Rubella	*Rubella virus*	Droplet transmission	768	196	21	-74.5***	-97.3***	-89.3***
Mumps	*Mumps virus*	Droplet transmission	8645	7392	6007	-14.5***	-30.5***	-18.7***
Influenza	*Influenza virus A and B*	Droplet transmission	8286	24423	342982	194.7***	4039.3***	1304.3***
Malaria	*Plasmodium spp.*	Vector-borne (mosquito bites)	309	123	344	-60.2***	11.3	179.7***
Schistosomiasis	*Schistosoma spp.*	Contact with contaminated water	9	1	0	-88.9*	-100	-100
Leptospirosis	*Leptospira spp.*	Contact with contaminated water/soil	77	2	1	-97.4***	-98.7***	-50
Syphilis	*Treponema pallidum*	Sexual transmission	16642	18904	26611	13.6***	59.9***	40.8***
Gonorrhea	*Neisseria gonorrhoeae*	Sexual transmission	4642	7299	7501	57.2***	61.6***	2.8
Brucellosis	*Brucella spp.*	Ingestion of contaminated dairy	240	708	1540	195***	541.7***	117.5***
Scarlet Fever	*Streptococcus pyogenes*	Droplet transmission	1839	1529	1630	-16.9***	-11.4***	6.6
Neonatal Tetanus	*Clostridium tetani*	Transmission through skin wound	12	2	0	-83.33*	-100	-100
Pertussis	*Bordetella pertussis*	Droplet transmission	59	389	197	559.3***	233.9***	-49.4***
Meningococcal Meningitis	*Neisseria meningitidis*	Droplet transmission	3	3	0	0	-100	-100
Typhoid and Paratyphoid	*Salmonella typhi*,* Salmonella paratyphi*	Fecal-oral transmission	2719	1075	821	-60.5***	-69.8***	-23.6***
Tuberculosis	*Mycobacterium tuberculosis*	Airborne transmission	30188	28353	24821	-6.1***	-17.8***	-12.5***
Dysentery	*Shigella spp.*,* Entamoeba histolytica*	Fecal-oral transmission	3985	1927	1072	-51.6***	-73.1***	-44.4***
Cutaneous Anthrax	*Bacillus anthracis*	Contact with infected animals/products	7	2	3	-71.4	-57.1	50
Dengue Fever	*Dengue virus*	Vector-borne (mosquito bites)	2040	263	13485	-87.1***	561***	5027.4***
Japanese Encephalitis	*Japanese encephalitis virus*	Vector-borne (mosquito bites)	110	31	16	-71.8***	-85.5***	-48.4*
Rabies	*Rabies virus*	Bite/saliva of infected animals	41	4	2	-90.2***	-95.1***	-50
Hemorrhagic Fever	*Hantavirus*,* Ebola virus*, others	Various (rodents, direct contact)	246	269	388	9.3	57.7***	44.2***
Measles	*Measles virus*	Airborne transmission	412	68	52	-83.5***	-87.4***	-23.5
Unspecified Hepatitis	Unknown	Varies	98	20	14	-79.6***	-85.7***	-30
Hepatitis E	*Hepatitis E virus*	Fecal-oral transmission	1423	1427	1226	0.3	-13.8***	-14.1***
Hepatitis D	*Hepatitis D virus*	Bloodborne transmission	2	2	2	-2.2	-2.2	-1.1
Hepatitis C	*Hepatitis C virus*	Bloodborne transmission	10752	10291	9973	-4.3**	-7.2***	-3.1*
Hepatitis B	*Hepatitis B virus*	Bloodborne transmission	19367	16364	15542	-15.5***	-19.8***	-5***
Hepatitis A	*Hepatitis A virus*	Fecal-oral transmission	1040	1148	626	10.4*	-39.8***	-45.5***
HIV/AIDS	*Human Immunodeficiency Virus (HIV)*	Sexual transmission, bloodborne	5699	4613	2954	-19.1***	-48.2***	-36***

*P < 0.05; **P < 0.01; ***P < 0.001

To ensure comparability across periods, we relied on the Yunnan CDC’s centralized surveillance network, which has maintained stable reporting standards over the past decade. This system is further supported by the Chinese Pathogen Identification Net (China PIN), a national laboratory network for pathogen surveillance and confirmation [8]. While pandemic-related factors—such as workforce strain, healthcare avoidance, or delayed reporting—may have influenced case reporting, case definitions and laboratory confirmation protocols remained unchanged across all three phases, helping to mitigate systematic bias. However, we acknowledge that potential shifts in diagnostic throughput or case prioritization during high-pressure periods may have introduced minor distortions in case ascertainment. This limitation is discussed further in Section “Limitations”.

### Statistical analysis

For each of the 36 infectious diseases included in this study, the mean monthly reported case counts for each phase were calculated to facilitate standardized comparisons across periods of differing lengths. To assess changes in reported case counts across the three pandemic-related phases, Poisson regression models were applied, with overdispersion assessed using the deviance-to-degrees-of-freedom ratio. Where overdispersion was detected, quasi‑Poisson or negative binomial models were used instead. All models included offset terms for phase duration and, where applicable, population size, to adjust for differences in exposure time or demographic scale. These models tested the null hypothesis that case counts did not differ significantly between time periods [9–11].

To quantify pandemic-related shifts and postpandemic rebounds, we computed three key case count ratios for each disease: (1) Pandemic suppression ratio: the ratio of mean monthly reported cases during 2020–2022 to those from 2014 to 2019, capturing the extent of reduction associated with NPIs. (2) Postpandemic resurgence ratio: the ratio of reported cases in 2023 to the average monthly case count for 2020–2022, reflecting rebound trends following policy relaxation. (3) Longitudinal shift ratio: the ratio of 2023 cases to those from 2014 to 2019, assessing the net change in disease burden over the full observation window.

95% confidence intervals for these ratios were estimated using nonparametric bootstrapping with 1,000 resamples, implemented via the boot package in R. This approach allows for robust inference when distributional assumptions of count data models may be violated. Resampling was performed on the observed monthly case counts, and the percentile method was used to derive confidence intervals.

To assess whether diseases that experienced greater suppression during the pandemic phase were more likely to exhibit resurgence post-pandemic, we calculated the Spearman rank correlation coefficient between the suppression ratio (2020–2022 vs. 2014–2019) and the resurgence ratio (2023 vs. 2020–2022) across all 36 diseases. In addition, linear regression models were fit to quantify the direction and strength of this association, using log-transformed ratios where appropriate. Influential observations were evaluated via leverage values and Cook’s distance, and multiple hypothesis testing was addressed using False Discovery Rate (FDR) correction to control for inflated type I error. Statistical significance was defined as *P* < 0.05.

All statistical analyses were performed in R version 4.3.3. Poisson, quasi‑Poisson, and negative binomial models were fit using the MASS package; correlation and linear modeling employed the stats package; bootstrapping used the boot package; and all visualizations were produced with ggplot2. All analysis scripts and data summaries are available in the supplementary materials or upon reasonable request.

### Ethics statement

This study utilized anonymized, aggregate-level surveillance data collected by the Yunnan Provincial Center for Disease Control and Prevention under China’s Infectious Disease Control Law. No personally identifiable information was accessed or analyzed, and individual consent was not required. Because the data were collected for routine public health surveillance and anonymized prior to analysis, this study was deemed exempt from institutional ethical review, in accordance with national guidelines and international norms for public health surveillance research. All procedures complied with relevant national regulations and standards for data confidentiality and privacy.

### Limitations

The use of COVID-19 case counts as a proxy for intervention intensity may not adequately reflect the specific effects of individual non-pharmaceutical measures. Additionally, potential inconsistencies in data collection and variations in healthcare-seeking behavior across different phases could have introduced comparability issues. Regional differences in healthcare infrastructure and public response patterns may further constrain the generalizability of the findings beyond Yunnan Province.

Another important limitation concerns potential underreporting of infectious diseases during the COVID-19 pandemic. This underreporting could have resulted from strained health systems, reduced diagnostic access, and decreased utilization of healthcare services by the public. Although consistent diagnostic protocols and mandatory reporting requirements were upheld by the Yunnan CDC throughout the study period, pandemic-related disruptions — including resource redirection toward COVID-19 testing, staff shortages, and operational burdens on sentinel surveillance networks — may have compromised routine disease reporting.

While no formal public assessment of surveillance completeness was available during the pandemic, the Yunnan CDC conducts internal data quality audits and adheres to national standards for surveillance accuracy, providing some reassurance regarding data consistency. Nonetheless, underreporting during high-intensity phases of the pandemic remains a plausible source of bias, underscoring the need for future research that integrates metrics on healthcare utilization and performance indicators from sentinel surveillance systems.

## Results

### Epidemiology of COVID‑19 in Yunnan Province (2020–2023)

Between January 2020 and December 2023, Yunnan Province experienced five distinct waves of COVID-19, reflecting shifts in public health interventions and policy changes.

The initial wave in early 2020 featured relatively low incidence, with peaks in January (91 cases) and February (83 cases). Case counts dropped sharply following the implementation of NPIs, including lockdowns, travel restrictions, and mass testing. For the remainder of 2020, only sporadic increases were reported in July (6 cases) and December (10 cases).

In 2021, moderate increases coincided with the partial relaxation of NPIs and seasonal transmission. Monthly peaks were observed in July (343 cases), August (270 cases), and September (329 cases), though overall incidence remained comparatively low.

A sharp resurgence occurred in late 2022 following the progressive easing of control policies. Monthly case numbers escalated in December, reaching 3,838 cases.

The largest wave emerged in 2023 after COVID-19 was downgraded to a Category B notifiable disease. The complete lifting of control measures was followed by a peak in June (103,000 cases), corresponding with resumed economic and social activity.

**Fig. 1:**
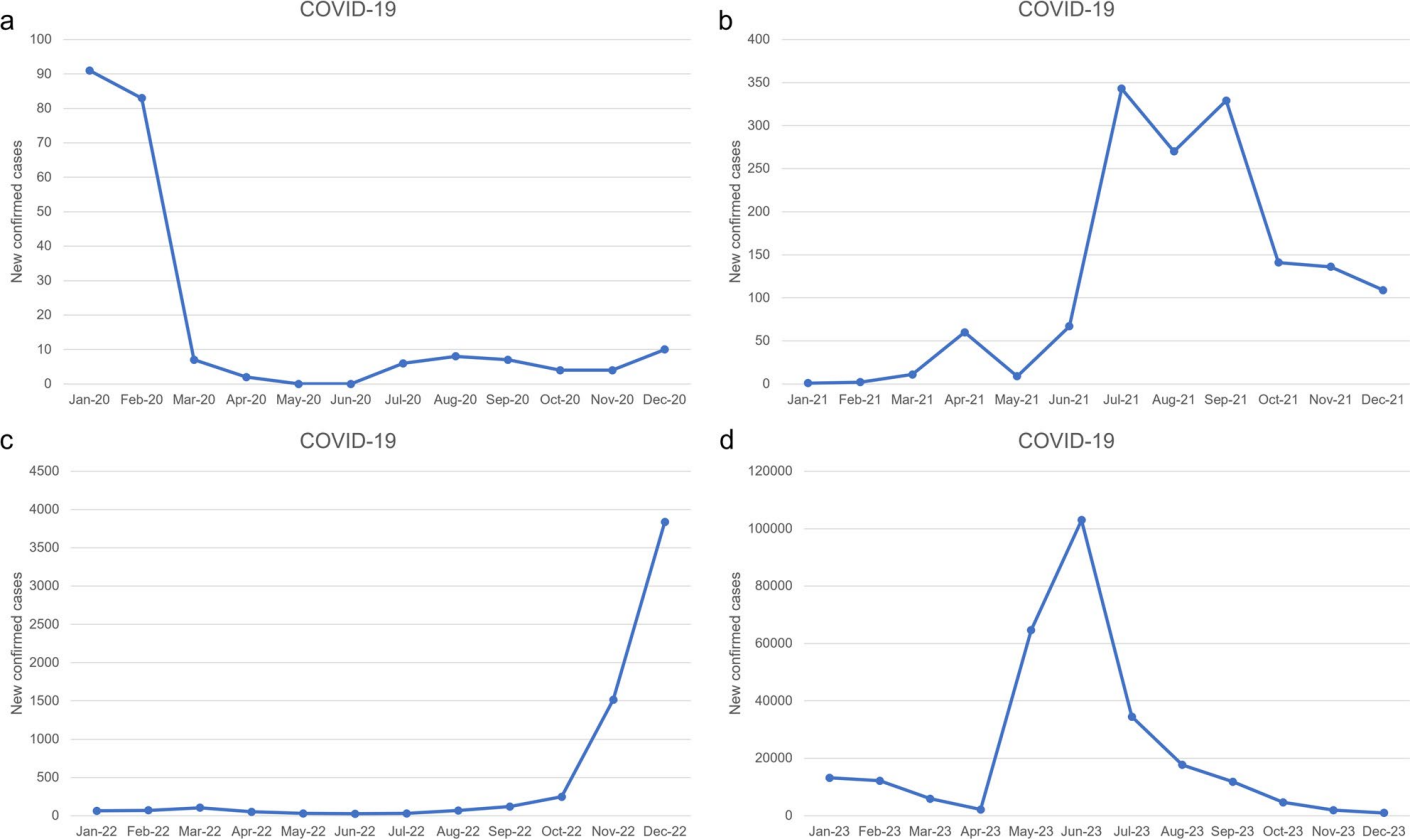
Monthly confirmed COVID-19 cases in Yunnan Province, China (January 2020–December 2023). Five distinct waves are observed, with peak incidence aligning with major policy transitions. The largest surge occurred in June 2023 following the relaxation of control measures and reclassification of COVID-19 as a Category B infectious disease

Subsequent months saw a rapid decline in reported cases, potentially indicating partial herd immunity or stabilization of transmission. A detailed timeline of monthly case counts alongside corresponding policy phases is provided in Fig. 1 and Supplementary Table S1.

### Impact of nonpharmaceutical interventions on infectious disease trends during the COVID-19 pandemic phase (2020–2022)

During the COVID‑19 pandemic phase (2020–2022), among the 36 notifiable infectious diseases included in this study, 24 experienced declines in reported case counts relative to the prepandemic baseline (2014–2019), of which 21 were statistically significant (*P* < 0.05). These reductions suggest broad inhibitory effects of NPIs on diverse transmission pathways (Table 1).

These reductions spanned multiple transmission pathways. Zoonotic infections exhibited the most marked declines, including leptospirosis (− 97.4%, ratio: 0.026, *P* < 0.001) and rabies (− 90.2%, ratio: 0.089, *P* < 0.001). Among vector-borne diseases, dengue fever declined by 87.1% (ratio: 0.129, *P* < 0.001), while schistosomiasis—a key waterborne parasitic infection—fell by 88.9% (ratio: 0.107, *P* < 0.05). For airborne vaccine-preventable diseases, measles (− 83.5%, ratio: 0.164, *P* < 0.001), rubella (− 74.5%, ratio: 0.256, *P* < 0.001), and mumps (− 14.5%, ratio: 0.855, *P* < 0.001) all showed notable decreases, likely reflecting changes in interpersonal contact patterns.

Diseases with sustained baseline transmission exhibited more modest declines. For instance, tuberculosis, a respiratory infection, declined by 6.1% (ratio: 0.939, *P* < 0.001); mumps, a vaccine-preventable disease with mixed transmission routes, by 14.5% (ratio: 0.855, *P* < 0.001); and HIV/AIDS, a bloodborne and sexually transmitted infection, by 19.1% (ratio: 0.809, *P* < 0.001). These patterns may reflect limited disruption of their endemic transmission dynamics during the pandemic period.

Despite these widespread declines, 12 infectious diseases showed increased case counts, with 10 reaching statistical significance (*P* < 0.05). The most marked increase was recorded for pertussis (+ 559.3%, ratio: 6.63, *P* < 0.001). Additional increases were observed for brucellosis (+ 195.0%, ratio: 2.95, *P* < 0.001), influenza (+ 194.7%, ratio: 2.95, *P* < 0.001), visceral leishmaniasis (+ 200.0%, ratio: 3.00, *P* < 0.01), echinococcosis (+ 91.7%, ratio: 1.92, *P* < 0.01), gonorrhea (+ 57.2%, ratio: 1.57, *P* < 0.001), and acute hemorrhagic conjunctivitis (+ 60.5%, ratio: 3.72, *P* < 0.001). These upward trends were primarily concentrated in the later years of the pandemic period.

In summary, the pandemic phase revealed a divergent pattern, with substantial reductions in many notifiable diseases alongside increases in others. These observations reflect the heterogeneous effects of population-level interventions on disease-specific reporting patterns.

### Postpandemic rebound in infectious diseases: 2023 resurgence

The evaluation of infectious disease trends across the prepandemic, COVID-19 pandemic, and postpandemic phases highlighted the varied impacts of public health interventions. Correlation analysis using ratios of reported cases from 2020 to 2022 compared with 2014–2019 and 2023 versus 2020–2022 revealed diverse trajectories of disease resurgence following the relaxation of COVID-19-related measures.

**Fig. 2:**
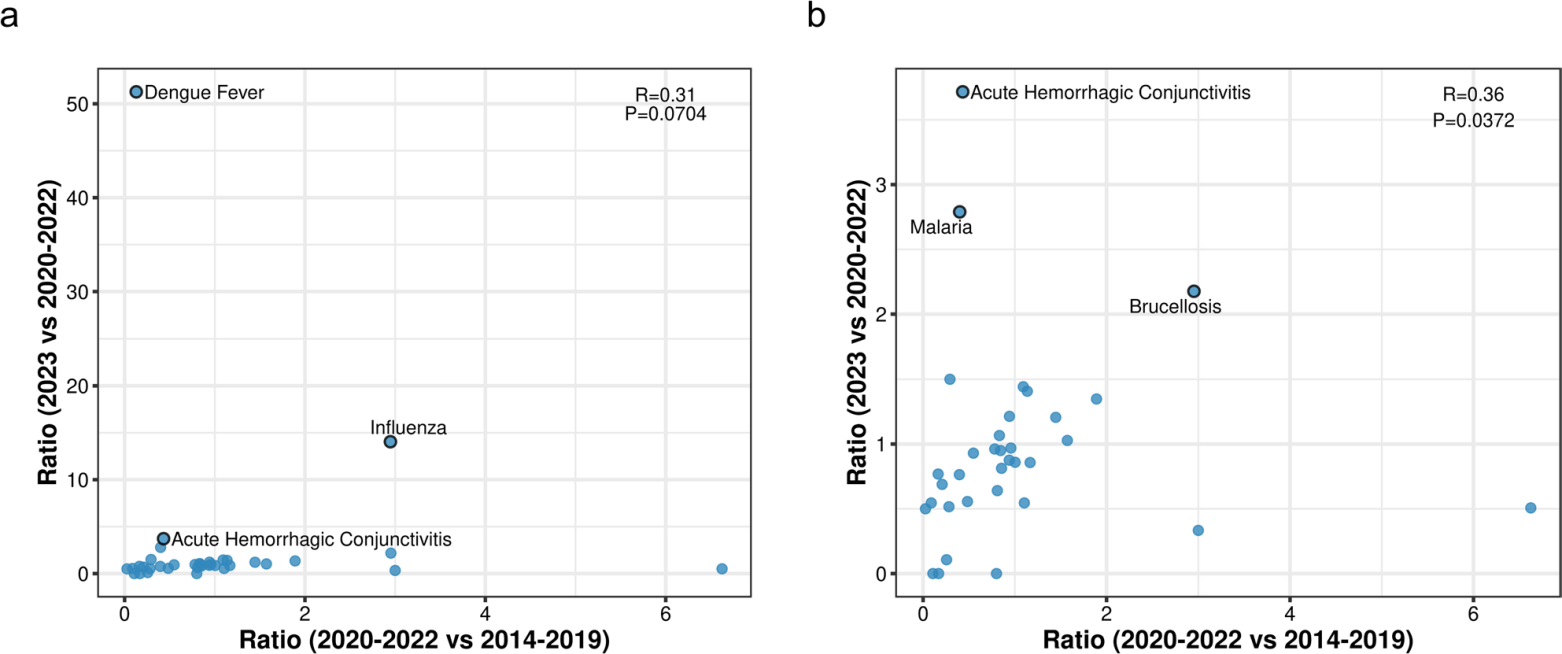
**a-b** Correlation analyses of infectious disease resurgence following the relaxation of COVID-19-related nonpharmaceutical interventions (NPIs). (**a**) Scatterplot showing the relationship between disease suppression during the COVID-19 pandemic phase (2020–2022 vs. 2014–2019) and resurgence in 2023 (2023 vs. 2020–2022) across 36 infectious diseases. A weak positive correlation was observed (*R* = 0.31, *P* = 0.0704), indicating substantial variability in post-NPI rebound. (**b**) After excluding outliers (dengue fever and influenza), the correlation became stronger and statistically significant (*R* = 0.36, *P* = 0.0372), suggesting more consistent resurgence patterns. Each point represents a disease; axes represent the respective incidence ratios

The initial scatterplot analysis (Fig. 2a) showed a weak positive correlation (*R* = 0.31, *P* = 0.0704), which was not statistically significant, indicating substantial variability in how diseases rebounded after the pandemic. After excluding high-leverage outliers such as dengue fever and influenza, the refined analysis revealed a significant correlation (*R* = 0.36, *P* = 0.0372; Fig. 2b), suggesting more predictable resurgence trends among a subset of diseases shaped by restored transmission pathways.

Following the lifting of NPIs in 2023, a subset of infectious diseases exhibited a notable resurgence. In total, 13 infectious diseases demonstrated increased case counts relative to 2020–2022, with 9 showing statistically significant increases (*P* < 0.05). These included diseases transmitted via vector-borne, respiratory, contact, zoonotic, and sexually transmitted routes, reflecting a broad reactivation of suppressed transmission dynamics.

**Fig. 3:**
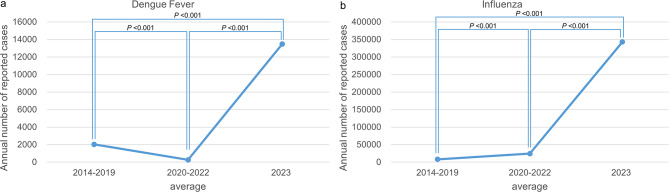
**a‒b** Trends in reported cases of dengue fever and influenza across the prepandemic, COVID-19 pandemic, and postpandemic phases in Yunnan Province. (**a**) Dengue fever: Case numbers declined significantly during the COVID-19 pandemic phase (2020–2022) compared to the prepandemic baseline (2014–2019), followed by a sharp resurgence in 2023 (*P* < 0.001). (**b**) Influenza: A similar suppression-rebound pattern was observed, with significant increases in 2023 (*P* < 0.001) post-NPI relaxation. Statistical differences were assessed using Poisson regression models. These results illustrate the impact of NPIs on transmission dynamics and the role of ecological and immunity-related factors in postpandemic resurgence

The most dramatic rebound was observed in dengue fever, with a surge of + 5027.4% (ratio: 51.27, *P* < 0.001), followed by influenza (+ 1304.3%, ratio: 14.04, *P* < 0.001), as shown in Fig. 3. Both diseases exhibited sharply elevated case counts in 2023 compared with the pandemic period, returning to or surpassing their prepandemic levels.

Other notable increases included acute hemorrhagic conjunctivitis (+ 271.5%, ratio: 3.72, *P* < 0.001), malaria (+ 179.7%, ratio: 2.79, *P* < 0.001), brucellosis (+ 117.5%, ratio: 2.18, *P* < 0.001), hemorrhagic fever (+ 44.2%, ratio: 1.44, *P* < 0.001), and typhus (+ 21.5%, ratio: 1.21, *P* < 0.01). The monthly case distributions (Table S1) showed that these increases were not limited to a single outbreak but occurred gradually over the course of the year, with higher intensities in mid to late 2023.

In contrast, 23 diseases maintained stable or reduced incidence levels in 2023 compared with 2020–2022. Among them, 13 diseases exhibited statistically significant declines. The most pronounced decreases were seen in rubella (− 89.3%, ratio: 0.11, *P* < 0.001) and pertussis (− 49.4%, ratio: 0.51, *P* < 0.001), indicating continued suppression of transmission despite the resumption of normal activities.

Further reductions were observed in fecal–oral and vector-borne diseases, such as dysentery (− 44.4%, ratio: 0.56, *P* < 0.001), hepatitis A (− 45.5%, ratio: 0.55, *P* < 0.001), and Japanese encephalitis (− 48.4%, ratio: 0.52, *P* < 0.05). Tuberculosis (− 12.5%, ratio: 0.88, *P* < 0.001) and HIV/AIDS (− 36.0%, ratio: 0.64, *P* < 0.001) also reported continued declines, possibly reflecting sustained public health programs.

Lastly, diseases such as leptospirosis and schistosomiasis remained at minimal levels in 2023, consistent with long-term suppression trends attributed to pandemic-era interventions.

## Discussion

The COVID-19 pandemic has profoundly reshaped infectious disease epidemiology worldwide through the widespread implementation of NPIs. In Yunnan Province, China, we examined trends in 36 notifiable infectious diseases across three distinct phases: prepandemic (2014–2019), pandemic (2020–2022), and postpandemic (2023). Our findings reveal a dual impact of NPIs: while they substantially suppressed many transmissible infections during the pandemic, their relaxation exposed latent rebound potential in multiple disease categories.

During the pandemic phase, stringent NPIs—such as travel restrictions, mandatory masking, and physical distancing—were temporally associated with notable declines across various disease types. In Yunnan, tuberculosis incidence declined by approximately 12.5%, while rubella cases dropped sharply by 74.5%, likely due to reduced interpersonal contact and improved respiratory hygiene practices. Similar reductions in respiratory infections were observed internationally, with up to 55% declines reported in South Korea and a near-elimination of seasonal influenza activity in Bavaria, Germany [3, 4].

NPIs also produced collateral benefits for gastrointestinal and waterborne infections. In China, several provincial studies documented significant declines in acute diarrheal disease incidence during periods of high NPI intensity [12, 13], and a multicenter analysis across sentinel hospitals reported suppressed detection rates for enteric pathogens during the same period [14]. In Yunnan, schistosomiasis and typhoid/paratyphoid fever declined by 88.9% and 60.5%, respectively, highlighting the contribution of mobility restrictions, enhanced sanitation infrastructure, and behavioral changes such as hand hygiene. These findings align with reports from Italy, where reductions in pediatric gastrointestinal infections were linked to COVID-era hygiene campaigns and school closures [15]. Nevertheless, gastrointestinal disease trends were not uniform across regions; for example, South Korea reported stable or even increased rates of some enteric infections during the pandemic [4], suggesting context-specific differences in NPI uptake and effectiveness.

During the postpandemic phase (2023), the relaxation of NPIs coincided with a pronounced resurgence of some infectious diseases. Vector-borne diseases, in particular, experienced striking rebounds: dengue fever surged by + 5,027.4% and malaria by + 179.7%. In Southeast Asia - including the Philippines and Malaysia - dengue case counts likewise exceeded prepandemic baselines [16, 17].

This resurgence likely reflects the convergence of multiple interacting drivers. First, vector control programs—such as community spraying and larval source reduction—were interrupted during the pandemic, weakening infrastructure and surveillance continuity. Second, the resumption of international travel may have introduced novel viral serotypes or variants, facilitating wider transmission. Third, favorable environmental conditions—such as increased rainfall, higher temperatures, and extended breeding seasons—amplified vector proliferation. For example, a study in Brazil applying interrupted time series methods demonstrated that dengue case numbers deviated substantially from expected trends during COVID-19, which authors attributed partly to disruptions in surveillance and public health responses [18]. Another ecological analysis in Brazil further documented a rebound in dengue burden postpandemic [19]. In Southeast Asia, spatiotemporal analyses in Malaysia, Thailand, and Singapore showed that COVID-related changes altered the relationship between dengue incidence and climatic factors [20]. These patterns underscore how deprioritizing endemic vector-borne disease control during a global health crisis can compound vulnerability and precipitate larger rebounds.

During the postpandemic phase (2023), zoonotic diseases also exhibited notable rebounds. Brucellosis incidence increased by 117.5%, likely reflecting resumed human–animal interactions in rural areas following the relaxation of COVID-19 control measures. Although veterinary services and livestock activities had largely resumed by 2023, temporary disruptions in animal health surveillance, reduced vaccination coverage, and decreased community outreach may have heightened exposure risks. Similar trends have been observed globally. In Central Asia and sub-Saharan Africa, economic pressures during the pandemic increased dependence on livestock and wildlife consumption, elevating the risk of zoonotic spillover [21]. The COVID-19 crisis exposed systemic gaps in zoonotic disease control and highlighted the need for an integrated One Health approach—linking human, animal, and environmental health sectors—to address these complex threats [22]. Understanding spillover dynamics is critical for designing effective early warning and surveillance systems [23]. The observed resurgence of brucellosis in our study underscores the urgency of cross-sectoral investment in zoonotic disease prevention and resilience building.

During the postpandemic phase (2023), respiratory diseases demonstrated heterogeneous patterns, with some diseases stabilizing while others experienced significant rebounds. In Yunnan Province, tuberculosis cases declined modestly by 12.5%, likely reflecting the continuation of diagnostic and treatment programs throughout the pandemic. This aligns with findings from Shantou and national-level analyses in China, where TB incidence decreased due to early detection efforts, improved infection control, and behavioral changes like masking during the pandemic period [24, 25]. However, time-series analyses suggest that this suppression was largely temporary, with TB incidence expected to return to baseline as health services normalize [26]. These findings emphasize the importance of sustained surveillance and cross-sectoral investment in TB programs beyond pandemic emergencies.

In contrast, influenza incidence sharply rebounded by + 1304.3% in 2023, coinciding with the withdrawal of NPIs such as universal masking and social distancing. This resurgence is consistent with global trends: in France, Reunion Island, and other regions, seasonal influenza and other respiratory viruses demonstrated strong rebounds after the relaxation of COVID-19 measures [27, 28]. In particular, recent studies have highlighted the asynchronous resurgence of respiratory viruses worldwide, including delayed but intensified waves of influenza, likely due to immune debt, behavioral normalization, and re-establishment of seasonal transmission cycles [29]. These observations underscore the need for enhanced preparedness, continued vaccine promotion, and targeted NPIs to mitigate respiratory disease burdens in the postpandemic era.

During the COVID-19 pandemic, routine pediatric health services were disrupted globally, potentially reducing immunization uptake and preventive care. In Yunnan Province, as in other regions, hand-foot-mouth disease (HFMD) and respiratory syncytial virus (RSV) experienced pronounced resurgences in the postpandemic period. These rebounds likely reflect immunological naivety among children, who were less exposed to circulating pathogens during extended periods of social restrictions. Comparable patterns were documented in Japan, where HFMD incidence surged following the reopening of childcare facilities [30], and in the United States, where RSV outbreaks intensified after the pandemic [31]. Similarly, in South Africa, substantial disruptions in child immunization programs have raised concerns about immunity gaps and increased vulnerability to vaccine-preventable diseases [32].

In Yunnan, rubella and mumps showed steep declines during the pandemic (− 89.3% and − 49.4%, respectively), likely due to reduced transmission driven by NPIs, such as school closures and social distancing. Although global vaccination programs were interrupted to varying extents, the observed declines may be more attributable to reduced person-to-person contact than to changes in vaccination coverage. This interpretation aligns with recent studies indicating that decreased incidence of respiratory and enteric infections during the pandemic was more strongly associated with NPIs than with immunization coverage disruptions [5, 6]. Postpandemic, modest rebounds in mumps may reflect residual immunity gaps, particularly in younger cohorts, and warrant continued surveillance. Given the absence of vaccination data in Yunnan, interpretations regarding immunization impact must remain cautious and context-specific. Globally, experts have emphasized the urgency of restoring and strengthening pediatric immunization programs to close immunity gaps and improve resilience against future health crises [33].

In this study, we observed sustained declines in environmentally mediated diseases, such as leptospirosis and schistosomiasis, during and after the COVID-19 period in Yunnan. These trends imply that public health interventions—especially those tied to WaSH (water, sanitation, hygiene)—may have had lasting suppressive effects on waterborne and parasitic transmission. Enhanced personal hygiene, greater community awareness, and limited environmental exposure due to constrained mobility likely contributed to these outcomes. Similar observations have been reported in South Asia and sub-Saharan Africa, where pandemic-era investments in WaSH infrastructure coincided with reduced diarrheal and parasitic disease burdens [34, 35].

Yet the 2023 resurgence of vector-borne diseases such as dengue and malaria reveals that ecological and entomological factors remain critical vulnerabilities. This contrast emphasizes not merely the gains made, but how fragile they are without sustained vector management and environmental health investment.

Our findings in Yunnan resonate with global experiences highlighting the need for context-specific adaptation. Japan’s strong NPI compliance produced substantial reductions in airborne disease transmission [30]. In the Caribbean, local adaptation of WHO COVID-19 guidelines improved resilience in outbreak settings [36]. In Nigeria, telemedicine and community-based engagement helped preserve essential health services under strain [37]. Similarly, decentralized rapid responses in Australian aged care settings demonstrated how tailored outbreak control can protect vulnerable populations [38].

We acknowledge several limitations. Reliance on notifiable disease data likely underestimates true disease burdens compared with sentinel or syndromic surveillance. Regional variation in surveillance capacity, healthcare access, and compliance with NPIs across Yunnan may constrain the generalizability of our findings [39, 40]. Furthermore, using COVID-19 case counts as a proxy for NPI intensity may obscure the distinct impacts of individual control measures. Future work should include granular intervention metrics, spatially resolved modeling, and cost-effectiveness evaluations to more precisely guide pandemic preparedness [41].

Finally, strengthening digital surveillance infrastructure—for example through SMS-based reporting, mobile apps, and web dashboards—can enhance real-time disease monitoring and response capacity, particularly in rural and resource-limited areas. Experience from SMS-based surveillance systems in low-resource settings has shown substantial improvements in reporting timeliness and completeness [42].

## Conclusions

The COVID-19 pandemic profoundly reshaped infectious disease dynamics, revealing both strengths and vulnerabilities within public health systems. In Yunnan Province, stringent NPIs significantly suppressed airborne and waterborne diseases, demonstrating their effectiveness in disrupting transmission pathways. However, the postpandemic resurgence of vector-borne and zoonotic infections underscores the challenge of sustaining such measures and highlights the need to address underlying ecological and social determinants of disease.

This study emphasizes the importance of maintaining robust surveillance systems and sustained investment in water, sanitation, and hygiene (WaSH) infrastructure to prevent future outbreaks. While One Health principles provide a comprehensive framework for managing zoonotic risks, practical efforts should focus on reinforcing context-specific, nonpharmaceutical prevention strategies, especially in settings with limited healthcare access.

As Yunnan transitions into the postpandemic phase, the lessons learned from the COVID-19 response may help guide adaptive public health strategies that strike a balance between immediate outbreak control and long-term health system resilience.

## Supplementary Information

Below is the link to the electronic supplementary material.

Supplementary Material 1: Supplementary Table 1. Monthly reported cases of 45 infectious diseases in Yunnan Province, China (2014–2023).

Supplementary Material 2: Supplementary Table S2. Annual reported cases of 36 infectious diseases in Yunnan Province, China (2014–2023).

Supplementary Material 3: Supplementary Figure 1 Annual reported cases of 36 infectious diseases in Yunnan Province, China, from 2014 to 2023, illustrating temporal trends across prepandemic, COVID-19 pandemic, and postpandemic phases.

## Data Availability

The raw data supporting the findings of this study are provided in Supplementary Table 1.

## Abbreviations

NPIs: Nonpharmaceutical interventions
SARS: Severe acute respiratory syndrome
LRTIs: Lower respiratory tract infections
HFMD: Hand-foot-and-mouth disease
RSV: Respiratory syncytial virus
WaSH: Water, sanitation, and hygiene
