# Supplementary figures and images for: Impact of nonpharmaceutical interventions on infectious disease patterns in Yunnan Province across the COVID-19 pandemic phases
Source: BMC Infect Dis. 2026 Jan 8;26:112. doi: 10.1186/s12879-025-12386-0 (PMC12825218; doi:10.1186/s12879-025-12386-0)

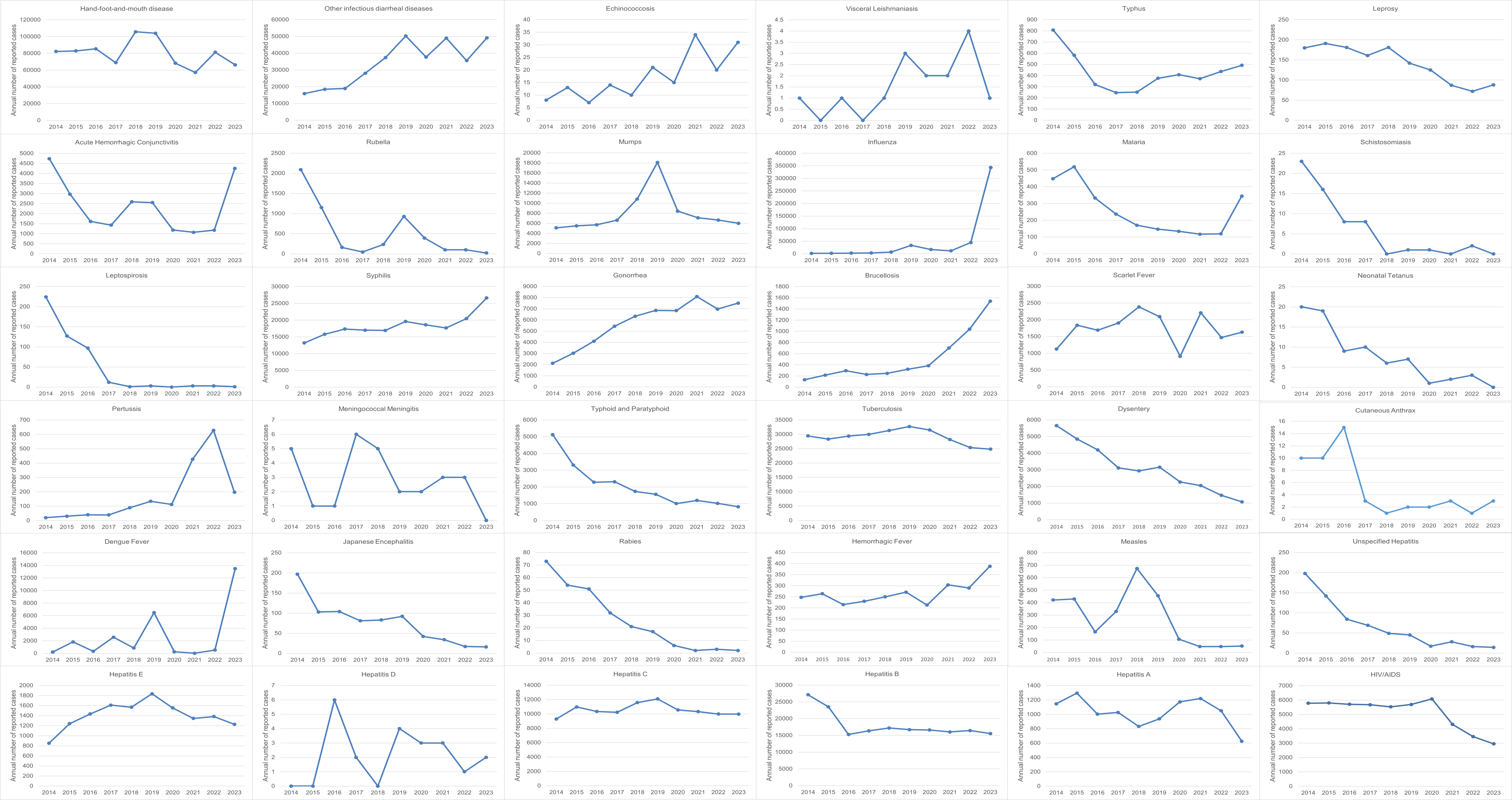

Supplement: Supplementary file 3 — Supplementary Material 3: Supplementary Figure 1 Annual reported cases of 36 infectious diseases in Yunnan Province, China, from 2014 to 2023, illustrating temporal trends across prepandemic, COVID-19 pandemic, and postpandemic phases. [file 12879_2025_12386_MOESM3_ESM.tif]
